# Effects of Walnuts on Postprandial Cognitive Function in Adults With Subjective Cognitive Impairment: Protocol for a Randomized Crossover Trial

**DOI:** 10.2196/82032

**Published:** 2025-12-19

**Authors:** Stephan Zarich, Rachel M Cole, Kelsey Fairchild, Daniel Spakowicz, Genevieve Sparagna, Annelise Madison, Ai Ni, Martha A Belury

**Affiliations:** 1 Department of Food Science and Technology The Ohio State University Columbus, OH United States; 2 Division of Medical Oncology The Ohio State University Columbus, OH United States; 3 Pelotonia Institute for Immuno-Oncology The Ohio State University Comprehensive Cancer Center Columbus, OH United States; 4 University of Colorado Anschutz Medical Campus Aurora, CO United States; 5 Department of Clinical Psychology University of Michigan Ann Arbor, MI United States; 6 Division of Biostatistics College of Public Health The Ohio State University Columbus, OH United States

**Keywords:** alpha linolenic acid, cognitive function, dietary fat, linoleic acid, polyphenols, postprandial meal challenge, subjective cognitive impairment, walnuts

## Abstract

**Background:**

Subjective cognitive impairment (SCI), the earliest sign of cognitive decline, affects 1 in 9 Americans aged older than 45 years. It negatively affects quality of life and is a risk factor for dementia. Healthy eating is a primary preventative strategy to impede cognitive decline. In the short term, cognitive function may be impacted by the consumption of a single meal, suggesting that the meal components, and not solely the metabolic dysregulation resulting from the condition of obesity, can impact cognition. The effect of meals on postprandial cognitive function is influenced by their macronutrient composition. A meal with a low-quality fat composition can acutely impair postprandial cognitive function. Walnuts are a source of high-quality fat as well as polyphenols. Some randomized control trials have shown that walnuts may benefit cognitive function. However, it is not clear whether a single meal high in walnuts can improve cognition in adults with SCI.

**Objective:**

The primary objective of the Essential Fats for Enhancing Cognitive Thinking study is to determine the impact of walnuts on postprandial cognitive function in adults with SCI. Secondary objectives include assessing the impact of daily walnut consumption for 1 week on cognitive function and erythrocyte fatty acids. Exploratory objectives include understanding the effect of walnut consumption on microbiota and intestinal inflammation.

**Methods:**

In this 7-week single-blind randomized crossover design study, 78 adults with SCI consumed 1 study snack per day, consisting of walnuts or a chocolate-style snack for 1 week, with a 4-week washout period between. Before consuming each study snack, participants underwent a meal challenge that included this study’s snack. Before randomization, participants completed a 1-week run-in period to become acclimated to consuming 1 study snack per day. A registered dietitian nutritionist counseled participants on incorporating this study’s snack into their diet while maintaining their body weight. Participants were blinded to which snack was the treatment and which was the control. Dietary intake and physical activity were measured with 24-hour recalls. Cognitive function was measured using the National Institutes of Health Toolbox for the Assessment of Neurological and Behavioral Function Cognitive Battery, both pre- and postprandially, as well as after 1 week of study snack consumption. Stool samples were collected weekly, except during the washout period, to measure microbiota α-diversity, β-diversity, and butyrate. Additionally, fasting blood samples, weight, and waist circumference were obtained at each study visit.

**Results:**

Recruitment began in February of 2024 and was completed by May 31, 2025.

**Conclusions:**

Improving cognition through the consumption of walnuts may ultimately prove to be an effective way to mitigate SCI.

**Trial Registration:**

ClinicalTrials.gov NCT06223672; https://clinicaltrials.gov/study/NCT06223672

**International Registered Report Identifier (IRRID):**

DERR1-10.2196/82032

## Introduction

### Background

Cognitive decline ranges from subjective cognitive complaints to mild cognitive impairment to diagnosed dementia. Subjective cognitive impairment (SCI) is the earliest sign of cognitive decline and consists of the experience of worsening or more frequent confusion or memory loss in the absence of observable functional decline [1]. Affecting 1 in 9 Americans aged older than 45 years, SCI is associated with mental distress and poses a risk for dementia [2-4]. Most people with SCI do not progress to a diagnosis of dementia, but some do. In a large US longitudinal cohort study, subjective cognitive decline occurred 4.4 years before mild cognitive impairment, 6.8 years before Alzheimer disease, and 6.9 years before all-cause dementia [5]. Even though the polygenic risk score did not differ between those with subjective cognitive decline and those without it, the former had more than double the risk of developing dementia (hazard ratio 2.14, 1.44-3.18). There are currently no treatments available for dementia; therefore, focusing on prevention strategies at the earliest sign of increased risk—when subjective complaints are present—is important [6].

Healthy eating is a primary preventative strategy for impeding cognitive decline [7,8]. The DASH (Dietary Approaches to Stop Hypertension) diet has been associated with a lower prevalence of subjective cognitive complaints [9]. Additionally, the Mediterranean-style diet and the Mediterranean–Dietary Approaches to Stop Hypertension Intervention for Neurodegenerative Delay diet have been associated with slower cognitive decline in older adults [10-12]. Furthermore, higher Mediterranean diet adherence has been associated with lower odds of having SCI [13,14]. The Mediterranean-style diet features foods rich in unsaturated fats, including sources of the essential fatty acids, α-linolenic acid (ALA) and linoleic acid (LA), as well as polyphenols such as flavonoids and tannins.

Chronic intake of saturated fat, on the other hand, is associated with poorer cognition and the risk for cognitive decline [15-17]. Even in the shorter term, 4 to 7 days, consumption of a high saturated fat diet can affect cognitive function [18-21]. Shorter still, the fat content of a single meal may impact cognitive function [22-28]. In adult males and females, after consuming a meal high in saturated fat, cognitive function is negatively impacted compared to a meal high in unsaturated fats [22,23]. Polyunsaturated fatty acids, on the other hand, may have a beneficial effect on cognitive function [29]. As a rich source of the polyunsaturated fatty acids ALA and LA, consumption of walnuts is associated with a lower risk of cognitive decline [30] and can influence postprandial cognitive function. In healthy, young adults, a high-fat breakfast featuring walnuts improved executive function and initially decreased, but later in the day increased, memory recall compared to a breakfast featuring butter [28]. However, the effect of walnut consumption on postprandial cognition in adults with SCI has not been examined.

The exact mechanism for the potential beneficial effects of walnuts on cognitive function is not known. Potential mechanisms include altering the gut microbiota by increasing β-diversity and shifting relative bacterial populations [31,32], or altering inflammation through metabolism of ellagitannins found in walnuts, which are metabolized to form anti-inflammatory urolithins [33-35]. Additionally, ALA and LA have been shown to reduce endotoxemia and inflammation [36-40]. Conversely, saturated fats promote inflammation, which negatively impacts cognitive function [41,42]. Both saturated and unsaturated fatty acids cross the blood-brain barrier, but this is not always detrimental; in moderation, saturated fatty acids may promote brain health and development [43,44]. Even without crossing the blood-brain barrier, fatty acids can impact the brain via inflammation. Saturated fats, which closely resemble the lipid portion of the endotoxin lipopolysaccharide, stimulate signaling via toll-like receptor-4 [45], an effect that mimics lipopolysaccharide’s effect [46]. Activation of toll-like receptor-4 stimulates production of proinflammatory cytokines that cross the blood-brain barrier, which causes neuroinflammation [47]. Saturated fats can also promote inflammation in the gastrointestinal tract, which increases intestinal permeability. A permeable intestine allows bacterial components such as lipopolysaccharide to escape into the bloodstream. Both lipopolysaccharide and saturated fats are powerful provocateurs of the inflammatory response, shifting the microbial composition and leading to a potentially unstable, imbalanced state, which further exacerbates proinflammatory signaling, resulting in a vicious cycle [15]. Additionally, lipopolysaccharide has been negatively associated with cognitive function [15]. Therefore, both chronic and acute dietary fatty acid consumption may influence cognition.

### Objectives

The objectives of the Essential Fats for Enhancing Cognitive Thinking (EFFECT) study are to (1) determine the impact of walnuts on postprandial cognitive function, and (2) assess the impact of daily walnut consumption for 1 week on cognitive function, the microbiota, and intestinal inflammation in adults with SCI.

## Methods

### Study Design and Setting

The EFFECT study was a 7-week randomized single-blinded placebo-controlled crossover design study. This study consisted of a 1-week run-in period followed by two 1-week intervention periods separated by a 4-week washout period. All participants were asked to consume 1 study snack per day throughout this study, except during the washout period. After completing the run-in period, participants were randomly assigned to the order of snack consumption: control snack followed by walnut snack or walnut snack followed by control snack. All study visits took place at The Ohio State University (OSU).

### Recruitment

A total of 78 adults with SCI were planned to be recruited from the central Ohio area. Inclusion and exclusion criteria are reported in Textbox 1. Study flyers were placed around the OSU campus and off-campus sites that include community centers, libraries, restaurants, and coffee shops. Additionally, study flyers were distributed to outpatient medical clinics in the Columbus, Ohio area. Potential participants were sent information about this study through The Ohio State Wexner Medical Center’s MyChart, and those who agreed to release their information to the research team were prescreened for eligibility. Radio advertisements were placed to reach potential participants outside of the OSU community. Recruitment for this study included or was carried out via ResearchMatch, a national health volunteer registry that was created by several academic institutions and supported by the US National Institutes of Health as part of the Clinical Translational Science Award program. ResearchMatch has a large population of volunteers who have consented to be contacted by researchers about health studies for which they may be eligible.

**Inclusion and exclusion criteria.**

**Inclusion criteria**

- Subjective cognitive impairment assessed as a score of ≥4 on either item of the Kohli scale
- Age 40-75 years
- BMI ≥20 kg/m^2^

**Exclusion criteria**

- Montreal Cognitive Assessment score of <23
- Diagnosis of mild cognitive impairment or dementia
- Current diagnosis of or current treatment of cancer other than nonmelanoma skin cancer or chronic stable cancers
- Gastrointestinal or pancreatic diseases or disorders where consumption of this study’s snacks would be contraindicated or not tolerated, or nutrient absorption would be impaired
- Bariatric or gastric bypass surgery
- Hyperthyroidism diagnosis
- Food allergy or intolerance or dietary restrictions, or use of supplements or medications, where consuming study snacks is contraindicated
- Use of supplements high in linoleic acid or α-linolenic acid in the 4 weeks before enrolling
- Pregnancy and lactation
- Psychostimulant or nootropic medication use
- Current use of supplements or medications for weight loss or following a weight loss program
- Severe or uncontrolled autoimmune diseases (excluding rheumatoid arthritis, psoriasis, and lupus)
- Current or previous diagnosis of severe kidney failure (glomerular filtration rate <30), liver cirrhosis, and some pulmonary diseases (ie, chronic obstructive pulmonary disease and emphysema)
- Heart disease events (including stroke or heart attack) within the last 3 months before enrollment
- Alcohol or drug abuse

Before providing informed consent, this study’s team screened potential participants over the phone using this study’s screening questionnaire to confirm that participants were likely eligible for this study. Those who passed the phone screening questionnaire and preferred to enroll in this study provided written informed consent before completing an in-person screen. The in-person screen consisted of measuring height and weight to calculate BMI, and completion of the Montreal Cognitive Assessment. Participants who passed the in-person screen began this study.

### Study Timeline

**Figure 1:**
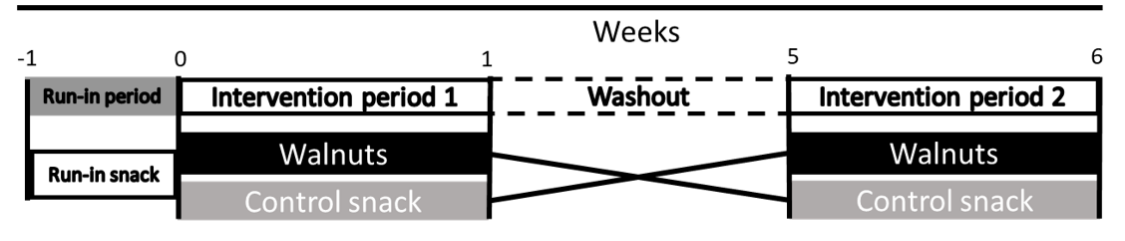
Study timeline.

The EFFECT study duration was 7 weeks and consisted of 5 study visits (Figure 1). Participants were asked to fast overnight for at least 12 hours before each study visit. Participants reported to the OSU campus for their in-person screening. Those who passed the in-person screen stayed and completed their first study visit (week –1), the start of the run-in period. During their week –1 visit, participants filled out the baseline questionnaire and listed their current medications and supplements. Blood pressure, pulse, temperature, and waist circumference were measured, as well as dominant hand grip strength. A finger stick and venous blood draw were also completed. The run-in period lasted 1 week and concluded with their week 0 study visit.

**Figure 2:**
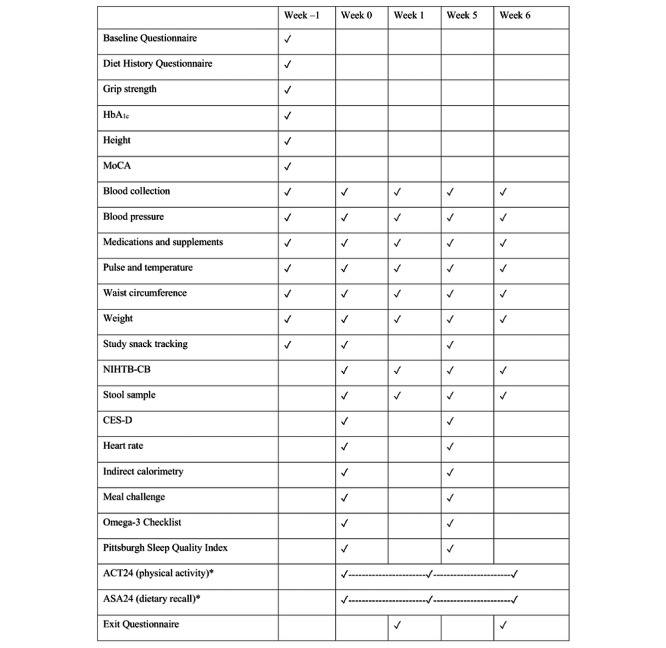
Study measurement timeline. ACT24: Activities Completed Over Time in 24 Hours; ASA24: Automated Self-Administered 24-Hour; CES-D: Center for Epidemiologic Studies Depression Scale; HbA1c: glycated hemoglobin; MoCA: Montreal Cognitive Assessment; NIHTB-CB: National Institutes of Health Toolbox for the Assessment of Neurological and Behavioral Function Cognitive Battery. *These were not administered at this study’s visits. They were given a total of 3 times during the study, which occurred between the week 0 visit and the week 6 visit. The intent is to capture snapshots of diet and activity during the beginning, middle, and end of the study. The “-----------------------" is meant to indicate that the measurement could occur at any time during this range.

At the beginning of each intervention period (weeks 0 and 5), participants reported to the Clinical Research Center on the OSU campus. At the Clinical Research Center, weight, vitals, and waist circumference were measured, sleep and diet questionnaires were answered, fasting indirect calorimetry was completed, a fasting blood draw was collected, and the meal challenge was administered with subsequent blood draws and indirect calorimetry measurements performed hourly for 5 hours. Additionally, cognitive function was measured both fasting and at 5 hours after the meal challenge, and any updates to medications and supplements were recorded. Each intervention period lasted 1 week and concluded with a study visit (week 1 and week 6). At the week 1 and week 6 visits, weight, vitals, and waist circumference were measured. A fasting blood draw was collected, cognitive function was measured, and updates to medications and supplements were obtained. There was a 4-week washout period between intervention periods. Figure 2 describes the measurements and information collected at each study visit. Participants were supplied with enough study snacks to consume 1 study snack per day until their next study visit. No study snacks were consumed during the washout period. At study visits, this study’s team verbally asked participants about adherence to consuming 1 study snack per day, changes to diet or activity, and changes to medications or supplements.

### Randomization

Participants were randomly assigned to the order of snack consumption: control followed by walnut or walnut followed by control. A statistician or graduate student studying statistics outside the clinical research team generated the randomization schemes in blocks of 2 and 4. After participants completed the week –1 study visit and before they completed their week 0 visit, the product coordinators assigned participants to the order of intervention from the randomization schemes. The product coordinators also prepared the snacks and meals for distribution to the participants, but had no interaction with the participants.

### Interventions

Participants were asked to consume 1 study snack per day throughout this study except during the washout period. Study snacks were portioned at the OSU research kitchen. At weeks –1, 0, and 5, participants were given enough portions of snacks to consume 1 portion per day until their next study visit. Participants were able to choose which time of day and whether to consume the whole portion at once or split it throughout the day. To avoid weight gain, this study’s dietitians worked individually with each participant to incorporate this study’s snack into their diet by replacing similar foods they were already consuming with this study’s snacks. All participants received instructions for incorporating this study’s snack into their diets and a list of typical foods to replace with this study’s snack. During the run-in period (week –1), participants consumed 1 study snack per day consisting of a brownie-style white chocolate snack (Eating Evolved; Table 1). During 1 intervention period, participants consumed a control snack consisting of a white chocolate-style bar (Eating Evolved). During the other intervention period, participants consumed walnuts.

**Table 1:** Study snack nutrition information^a^.

	1 Serving (g)	Energy (kcal)	Fat (g)	Saturated fat (g)	MUFA^b^ (g)	PUFA^c^ (g)	Carbohydrates (g)	Sugar (g)	Fiber (g)	Protein (g)
Run-in period snack	67	369	34	11	17	4	23	3	10	7
Walnuts	56	366	37	3	5	26	8	1	4	9
White chocolate-style bar	68	366	34	15	15	3	24	2	17	5
Alternate run-in snack	73	364	31	13	—^d^	—	29	3	10	8
Alternate control snack	75	375	35	18	—	—	25	3	7	10

^a^Monounsaturated fatty acid and polyunsaturated fatty acid information was not available for alternate snacks.

^b^MUFA: monounsaturated fatty acids.

^c^PUFA: polyunsaturated fatty acids.

^d^Not available.

Due to unforeseen supply chain issues, which eliminated access to snacks, an alternate run-in period snack consisting of chocolate-covered peanuts (Lakanto, Saraya USA, Inc) and an alternate control snack consisting of dark chocolate peanut butter cups (Lakanto) were used. Alternate snacks were chosen based on matching energy, macronutrients, and saturated fat to the original run-in and control snacks as closely as possible. All study snacks had no added sugar.

The nutrition information of this study’s snacks is provided in Table 1. Participants were provided with nutrition information on all snacks during this study.

During this study, participants were asked to maintain their normal physical activity and eating patterns except for replacing a similar habitual food with this study’s snack and removing foods high in ellagitannins and replacing them with similar foods low in ellagitannins.

Throughout this study, the study’s dietitians were available to help participants avoid weight gain from the study’s snacks. The goal was for participants to maintain their weight for the entire study. Weight maintenance was considered at ≤±2 kg over 2 weeks. This study’s dietitians discussed with the participant potential reasons for weight change and adjusted counseling as needed. Participants were able to choose which time of day they consumed this study’s snack, and whether it was consumed as part of a meal or as a snack. The participants chose whether to consume this study’s snack all at once or spread the portion out over the course of the day. The participants were asked to record the study snack they consumed each day and the relative time of day this study’s snack was consumed (morning, afternoon, evening, etc). Participants were asked to return uneaten study snacks at their next study visit.

### Blinding

The EFFECT study was single-blinded. Participants were not told which snack was the treatment and which was the control. The researchers told participants that 2 snacks were being investigated, but did not disclose the hypothesis of this study to participants. Due to the nature of this study, it was not possible to blind the research team. Once all participants completed this study, they received information about which study snack was the treatment and which study snack was the control. As the snacks were not blinded to the participants or the clinical study team, there was no testing of blinding success.

### Outcomes

#### Cognitive Function

The primary outcome of the EFFECT study is the difference in postprandial cognitive score between control and walnut interventions. Additionally, the difference in change of premeal score at the start and end of the intervention period between the control and walnut interventions will be analyzed. To assess cognitive function, the National Institutes of Health Toolbox for the Assessment of Neurological and Behavioral Function Cognitive Battery (NIHTB-CB) was used, specifically the fluid cognition battery. The tests are fully automated and administered via an iPad (Apple Inc) with consistent program-derived instructions. Minimal practice effects and no floor or ceiling effects are seen with the NIHTB-CB [48-50].

#### Microbiome

Participants were instructed to collect a stool sample on the 1-2 days before or the morning of week 0, 1, 5, and 6 study visits. An Omnigene Gut fecal collection kit (OMR-200, DNAGenotek) was provided with specific instructions for how to store the sample until delivery to the clinic. Total DNA will be isolated using QIAGEN Power Fecal Pro kits and sequenced to a depth of >25 million reads per sample. Microbe or gene abundances will be defined via alignment to reference genomes and de novo assembly.

#### Anthropometrics

Waist circumference was measured at each study visit using a nonstretch tape measure at the uppermost border of the right iliac crest. Weight and height were measured without shoes using a digital scale and a stadiometer, respectively. Weight was measured to the nearest 1 kg, and height was measured to the nearest 0.1 cm. Weight was measured at each study visit, and height was measured at the first study visit. Height and weight were used to calculate BMI. Grip strength was measured at the first study visit using a Jamar handheld dynamometer and reported in kilograms as previously described [51].

#### Meal Challenge

At weeks 0 and 5, participants received a high-kilocalorie meal challenge. The meal was approximately 900 kcal with 60 g of fat, 33 g of polyunsaturated fat for the walnut meal and 41 g of saturated fat for the control meal, 60 g of carbohydrates, and 37 g of protein; the meal consisted of a drink and the corresponding intervention period study snack. The macronutrient composition of the meals between the 2 study snacks was equivalent except for the saturated fat, monounsaturated fat, and polyunsaturated fat content. Participants had 20 minutes to consume the entire meal. To measure preprandial and postprandial metabolism, before consuming the meal (fasting) and each hour for 5 hours after consuming the meal, energy expenditure, blood pressure, and heart rate were measured, and venous blood samples and saliva samples were collected.

Energy expenditure was measured using indirect calorimetry (Ultima Series CPX, MGC Diagnostics). For the fasting indirect calorimetry, after arriving for their study visit, participants rested in a quiet, dim room on a hospital bed with the head of the bed at a 30° angle for 30 minutes before assessing energy expenditure over 25 minutes. For postprandial measurements, participants rested for 30 minutes in a hospital bed while being allowed to quietly read, use electronics, or sleep before a 20-minute indirect calorimetry measurement period. A heart rate monitor (FirstbeatTM Bodyguard 3, Firstbeat Technologies) was placed on the participant’s chest and abdomen at the start of the first resting period and measured their heart rate throughout this study’s visit. Fasting and postprandial blood pressure were measured using a blood pressure cuff.

To reduce variability in postprandial measurements, participants were provided with all meals and snacks for the day before the meal challenge [52]. As described in prior research, the meals and snacks were approximately 30% fat, 20% protein, and 50% complex carbohydrate with low added sugars [53,54] and were prepared by the Ohio State Clinical Research Center. To further reduce variability of postprandial measurements, participants were also instructed to avoid taking blood pressure, glucose-lowering, lipid or cholesterol-lowering, and nonsteroidal anti-inflammatory medications the morning of the meal challenge, to avoid alcohol the day prior, to avoid strenuous exercise 2 days prior, and to abstain from taking dietary supplements and aspirin for 7 days before the meal challenge.

#### Mitochondrial Measurements

Cardiolipin species will be measured in the peripheral blood mononuclear cell (PBMC) samples. PBMC cardiolipin fatty acyl molecular species mass will be quantified using electrospray ionization-mass spectrometry coupled to high-performance liquid chromatography as previously described [55,56].

#### Biochemical Markers

Glycated hemoglobin was measured using a finger stick. Erythrocyte and PBMC fatty acids will be analyzed. PBMC lipids will be extracted using methanol and chloroform (1:2 v/v) [57] and methylated using 5% hydrochloric acid in methanol [58] as previously described [59]. Erythrocyte fatty acid methyl esters will be prepared using 14% boron trifluoride in methanol and hexane [60] as previously described [61,62]. Gas chromatography with a 30-m Omegawax TM 320 fused silica capillary column (Supelco) will be used to analyze the fatty acid methyl esters. Standards for fatty acid methyl esters (Matreya, LLC and Nu-Check Prep Inc) will be used to determine retention times of fatty acids in the samples as previously described [63].

From blood collected at fasting and postprandial time points, lipid panels were measured at the OSU Wexner Medical Center Hospital Lab. Glucose will be measured using the Glucose Autokit (FUJIFILM Healthcare Americas Corporation), and insulin levels will be measured using the Meso Scale Diagnostics kit. Fasting glucose and insulin levels will be used to calculate estimated insulin resistance using the homeostatic model assessment of insulin resistance [64]. Markers of inflammation, including fasting and postprandial tumor necrosis factor alpha, tumor necrosis factor receptor 2, C-reactive protein, interleukin-6, and soluble intercellular adhesion molecule-1, will be measured at weeks 0 and 5. Fasting soluble CD14, oxidized low-density lipoprotein, and lipopolysaccharide binding protein will also be measured. Tumor necrosis factor α, tumor necrosis factor receptor 2, soluble intercellular adhesion molecule-1, C-reactive protein, interleukin-6, and lipopolysaccharide binding protein will be measured using a highly sensitive Meso Scale Diagnostics kit, and soluble CD14 and oxidized low-density lipoprotein will be measured by ELISA kits (R&D Systems and Mercodia, respectively). Lipopolysaccharide will be measured using Kinetic-QCL, Kinetic Chromogenic LAL Assay, or ELISA LifeSpan BioSciences. Markers of oxidative stress, 4-hydroxynonenal and malondialdehyde, will also be measured by multiplex methods as previously reported [54,65-68].

#### Questionnaires and Recalls

At the first study visit, participants were asked to complete the Baseline Questionnaire, a descriptive questionnaire asking about age, gender, sex, race, ethnicity, and health status. At the beginning of each intervention period, participants were asked to complete the Pittsburgh Sleep Quality Index [69] to capture sleep quality and disturbances, an Omega-3 Fatty Acid Checklist to determine omega-3 fat intake [70], and the Center for Epidemiologic Studies Depression Scale to assess depressive symptoms.

To measure dietary intake before enrollment in this study, participants completed the Diet History Questionnaire III, a food frequency questionnaire developed by the National Cancer Institute staff [71,72]. During this study, participants completed 24-hour dietary recalls, using the Automated Self-Administered 24-Hour Dietary Assessment Tool (version 2022 and 2024), developed by the National Cancer Institute. To assess changes in physical activity, 24-hour recalls were completed using the Activities Completed Over Time in 24 Hours, a web-based physical activity recall tool offered by the National Institutes of Health National Cancer Institute [73-75].

### Sample Size

The primary outcome used in sample size calculation was the postprandial cognitive test score from the NIHTB-CB. Based on our previous study [22], we obtained the SD (11.0) of the difference in postprandial cognitive test score between the unsaturated fat meal and the saturated fat meal. We had 80% power with 78 participants to detect a difference of 3.5 in postprandial cognitive test score between the control and the walnut group using a 2-sided paired *t* test with a type I error of 0.05.

### Statistical Analysis

Postprandial cognitive test scores will be compared between the control and walnut group using a 2-sided paired *t* test to account for the cross-over design. To further adjust for covariates, a linear mixed effect model with subject-specific random intercept will be fit on postprandial test score with the following covariates: study group, preprandial test score at week 0 and 6, BMI, age, sex, education level, and depression score. The regression coefficient of this study’s group will be the adjusted difference in postprandial test score between this study’s groups. Potential carryover of treatment effects from the first treatment period to the second period will be assessed by including an interaction between treatment and period in the mixed-effect models. Significant interaction indicates the existence of a carryover effect, in which case we will only use data from the first treatment period to evaluate the treatment effect using a 2-sample *t* test and linear regression models. Analyses for the primary hypothesis will use participants who completed the entire study and will be based on an intention-to-treat principle, regardless of compliance and study group crossover. Sensitivity analyses will be conducted with multiple imputation on missing values for participants who do not complete the entire study. A linear mixed effect model with subject-specific random intercept will be fit on preprandial test score at week 1 and 6 with the following covariates: study group, preprandial test score at day 0 and 35, BMI, age, sex, and education level. The regression coefficient of this study’s group will be the adjusted difference in preprandial test score after a 7-day intervention between study groups. A linear mixed effect model with subject-specific random intercept will be fit on postprandial test score at weeks 0 and 5 with the following covariates: change in plasma sum of ALA+LA during 7-day intervention, BMI, age, sex, and education level. The regression coefficient of plasma sum of ALA+LA will measure the adjusted correlation between postprandial test score and change in plasma sum of ALA+LA.

A linear mixed effect model with subject-specific random intercept will be fit on the change of endotoxemia during the 7-day intervention with the following covariates: study group, BMI, age, sex, and education level. The regression coefficient of this study’s group will be the adjusted difference in the reduction of endotoxemia between this study’s groups. β*-*diversity of the microbiome community at days 7 and 42 between all pairs of participants will be calculated using the Bray-Curtis dissimilarity measure. Nonmetric multidimensional scaling ordination will be computed, and nonmetric multidimensional scaling plots will be generated to visualize the difference in β-diversity between study groups. Permutation-based ANOVA will be used to compare the β-diversity between study groups. Operational taxonomic units (OTU) abundance will be compared between study groups using paired *t* tests for each OTU, and the *P* values will be adjusted by the Benjamini-Hochberg procedure [76] to control for false discovery rate. A linear mixed effect model with subject-specific random intercept will be fit on postprandial test score with the following covariates: change of endotoxemia during the 7-day intervention, change of OTU abundance during the 7-day intervention, BMI, age, sex, and education level. As the number of OTUs will be very large, exceeding the sample size, a LASSO (Least Absolute Shrinkage and Selection Operator) penalty [77] will be included in the regression model to select significant covariates.

### Ethical Considerations

Review and approval for this study and all procedures were obtained from the OSU Institutional Review Board on April 24, 2023 (2023H0111). All interested potential participants were provided verbal and written information about this study, including a copy of the consent form. Potential participants had the opportunity to ask questions and be provided as much time as necessary to review the consent form and consider participating. Informed consent was obtained from all participants. Participants were compensated up to US $200 for participation in this study, where those who discontinued early received a prorated amount. Efforts were made to protect the privacy of this study’s participants, such as deidentifying participant information and limiting interactions with participants to essential study team members.

## Results

The EFFECT study was funded in May 2023. Recruitment began in February 2024, and the final study visits were projected to be completed in July 2025.

## Discussion

### Comparison With Prior Work

Studies examining cognitive function after consumption of a meal are limited. Fewer still are reports on the impact dietary fat has on postprandial cognitive function [22-24,28]. Of those investigations, comparisons have been made between a saturated fat meal and an unsaturated fat meal [22,23]. The unsaturated fat meals used dietary oils that were mostly composed of monounsaturated fat. In a population of women, the high saturated fat meal had a negative impact on cognitive function compared to the high unsaturated fat meal [22]. In a population of men, the negative impacts of the high saturated fat meal were only observed in men who were obese [23]. In contrast, the EFFECT study did not use dietary oils and instead used walnuts to deliver the unsaturated fat, included both men and women, and included anyone with a BMI over 20.

Delivering unsaturated fats and polyphenols through nuts instead of oil is not novel. A meal supplemented with almonds has also been investigated [24]. This meal was compared to a high-carbohydrate meal, which found that the high-fat almond meal ameliorated declines in cognitive function 2 hours after the meal. Most of the fat in almonds is monounsaturated fat. Differing from almonds, walnuts contain a higher proportion of polyunsaturated fats. Of note are ALA and LA, the 2 essential polyunsaturated fatty acids that cannot be synthesized and therefore must come from the diet. Walnuts are a rich source of both ALA and LA, whereas almonds contain no ALA and only a fraction of the LA that is present in walnuts. The unique fat composition and polyphenols of walnuts may have different effects on postprandial cognitive function compared with the nutrient composition of almonds.

The EFFECT study is not the first to explore a high polyunsaturated fat meal using walnuts in relation to cognitive function. A recent crossover trial investigated the effects of a breakfast containing walnuts on cognitive performance [28]. After consuming the walnut breakfast, improvements to executive function were observed compared to a control meal featuring butter as the primary fat source. The effects on memory were mixed, observing a decrease in recall at 2 hours after the meal, but an increased recall at 6 hours compared to the control. This could be related to the walnuts slowing of gastric emptying, which could result in a delayed response. This design administered cognitive tests at baseline and 2, 4, and 6 hours after breakfast, which differs from the EFFECT study, as postprandial cognitive testing was only completed at hour 5. Furthermore, participants were given a snack before the baseline tests and given a lunch between hours 2 and 4. In the proposed study, participants fasted for baseline cognitive tests and did not consume additional food after the high-calorie breakfast. Additionally, the participants were young, healthy adults, which differs from the older population with SCI in the EFFECT study. Therefore, the proposed study would expand our understanding of postprandial cognitive function.

In addition, walnuts alter the gut microbiota by increasing β-diversity and shifting relative bacterial populations [31,32]. Walnuts are a source of ellagitannins, which are metabolized to form anti-inflammatory urolithins [33-35]. We will look at the relationship between gut microbiota, urolithins, and postprandial cognitive function.

### Strengths and Limitations

The EFFECT study uses a randomized, crossover design with a 4-week washout period between interventions. Thus, a strength of this design is that participants served as their own controls. A total of 4 weeks should be sufficient to prevent any carryover effects from the first intervention. Using a run-in period could be considered a limitation, as a study snack used to acclimate participants to consuming 1 study snack per day would be a deviation from their usual diet. However, the fat profile of the run-in period snack is comparable to the typical American diet. An inherent limitation to studying SCI is the subjective nature of the cognitive impairment, as reliance on a self-report of cognitive issues is necessary. Similarly, adherence to the interventions is partially reliant on self-reported consumption. However, changes in fatty acid profiles in erythrocytes were also used as a measure of adherence.

### Conclusions

With no current dietary recommendations for managing SCI, discovering a food that improves cognitive function in adults with SCI is important. The dietary interventions in this study are modest and only require substituting a study snack for a food already being consumed. Walnuts can be easily incorporated into an individual’s diet, and a total dietary overhaul is not required. Consuming certain fats, or reducing consumption of others, could be an effective, inexpensive way to impact the negative effects of SCI. The EFFECT study will enhance our knowledge of the interplay between cognitive function, dietary fats, and SCI.

## Abbreviations

ALA: α-linolenic acid
DASH: Dietary Approaches to Stop Hypertension
EFFECT: Essential Fats for Enhancing Cognitive Thinking
LA: linoleic acid
LASSO: Least Absolute Shrinkage and Selection Operator
NIHTB-CB: National Institutes of Health Toolbox for the Assessment of Neurological and Behavioral Function Cognitive Battery
OSU: Ohio State University
OTU: operational taxonomic unit
PBMC: peripheral blood mononuclear cell
SCI: subjective cognitive impairment

## Data Availability

The dataset is not publicly available due to the ongoing nature of the trial. Upon conclusion of the trial, data will be available from the corresponding author upon reasonable request.
